# Potential Contributions of Sleep and Circadian Rhythms to Behavioral Difficulties in Children with Smith–Magenis Syndrome in Real Life: An Actigraphy-Based Study

**DOI:** 10.3390/children13080987

**Published:** 2026-07-24

**Authors:** Marion Comajuan, Sabine Plancoulaine, Caroline Demily, Marie Noelle Babinet, Julien Lioret, Lisa Brunel, Leana Rivet, Carmen M. Schröder, Aurore Guyon, Patricia Franco

**Affiliations:** 1 Sleep Pediatric Unit, Service d’Epileptologie Clinique, des Troubles du Sommeil et de Neurologie Fonctionnelle de l’Enfant, Hospices Civils de Lyon, 69677 Bron, France; 2 Centre de Recherche en Neurosciences de Lyon CRNL U1028 UMR5292, Physiologie Intégrative des Systèmes D’éveil du Cerveau, Equipe Waking, CNRS, INSERM, Université Claude Bernard Lyon 1, 69500 Bron, Franceext-lisa.brunel@chu-lyon.fr (L.B.); leana.rivet@orange.fr (L.R.); 3 GénoPsy, Centre de Référence Maladies Rares Troubles du Comportement d’Origine Génétique (GénoPsy-Lyon), LeVinatier Psychiatrie Universitaire Lyon Métropole et UMR 5229, CRNS & Université Lyon 1, 95 Boulevard Pinel, 69678 Bron, Francemarie-noelle.babinet@ch-le-vinatier.fr (M.N.B.); 4 Pôle Hospitalo-Universitaire Autisme, Neurodéveloppement et Inclusion Sociale, Centre de Référence Maladies Rares GénoPsy, Centre d’Excellence iMIND, Le Vinatier Psychiatrie Universitaire Lyon Métropole, 69678 Bron, France; 5 Department of Child and Adolescent Psychiatry, Excellence Centre for Neurodevelopmental Disorders STRAS&ND, Centre for Rare Diseases with Psychiatric Expression, Strasbourg University Hospitals, Strasbourg University, 67091 Strasbourg, France; 6 CNRS UPR 3212, Institute for Cellular and Integrative Neurosciences, 67000 Strasbourg, France

**Keywords:** Smith–Magenis syndrome, circadian rhythms, actigraphy, questionnaire, children

## Abstract

**Highlights:**

**What are the main findings?**

- In children with Smith–Magenis syndrome, subjective sleep disturbances were significantly associated with behavioral difficulties, particularly irritability, stereotypic behaviors, and inappropriate speech.
- Objective circadian rhythm measures, especially earlier L5 onset, were associated with hyperactivity and social withdrawal, whereas no associations were found with standard actigraphy sleep continuity parameters.

**What are the implications of the main findings?**

- Exploratory analyses suggest that sleep quality and circadian rhythm characteristics are associated with different behavioral domains, which may reflect distinct underlying mechanisms.
- If confirmed in larger prospective studies, these findings may help identify sleep and circadian rhythm characteristics as potential targets for future interventions in children with Smith–Magenis syndrome (such as chronotherapy approaches).

**Abstract:**

**Background:** Smith–Magenis syndrome (SMS) is a rare neurodevelopmental disorder characterized by severe sleep disturbances, circadian rhythm dysregulation, and prominent behavioral difficulties. **Objective:** To examine associations between subjective and objective sleep and circadian characteristics and behavioral difficulties in children with SMS in real life. **Methods:** Children aged 5–13 years with genetically confirmed SMS were included in a prospective single-center cohort study. Sleep and circadian rhythms were assessed using parent-reported questionnaires (Sleep Disturbance Scale for Children (SDSC), Horne and Östberg Morningness–Eveningness Questionnaire (H&O)) and 15-day home-based wrist actigraphy. Behavioral difficulties were evaluated using the Aberrant Behavior Checklist-Community (ABC-C). Associations between subjective and objective sleep and circadian measures with behavioral difficulties were studied using Spearman correlations for each ABC-C subscale. **Results:** Among the 20 children included (55% female, mean age 10 ± 2.5 years), 75% were carriers of a 17p11.2 microdeletion and 25% had an *RAI1* mutation. Overall, 95% of children were treated with melatonin, 55% with psychostimulants and 20% with beta-blockers. According to the SDSC results, pathological sleep disturbances were present in 14/20 (70%) children and concerned mainly complaints of insomnia (10/20, 50%) and excessive daytime sleepiness (8/20, 40%). The H&O chronotypes confirmed the morning type in 70% of patients. Sleep and circadian measures obtained by actigraphy showed reduced total sleep time in 71% of the children, reduced sleep efficiency in 88%, long wake after sleep onset (WASO > 60 min) in 59%, as well as an early L5 onset (5 h period with the least movements < 23:38) and M10 onset (10 h period with the highest activity levels < 09:12) in 92% and 62% of children, respectively. The behavioral domains of the ABC-C with the highest scores were hyperactivity (mean 52.1, range 2.1–97.9) and irritability, agitation, and crying (mean 46.9, range 17.8–82.2). In the analysis of the relationship between behavior and sleep, a positive correlation was found between sleep disorders and stereotyped behaviors (r = 0.472, *p* = 0.048), insomnia and irritability, social withdrawal and stereotyped behaviors (r = 0.472, *p* = 0.048; r = 0.701, *p* = 0.001; and r = 0.648, *p* = 0.004, respectively), and non-restorative sleep and inappropriate speech (r = 0.476, *p* = 0.046). No significant association was found between objective sleep measures and behaviors. While subjective assessments suggested that a stronger morning chronotype was associated with lower irritability (r = −0.565, *p* = 0.015), actigraphy-derived circadian rhythm analyses revealed that an earlier L5 onset was associated with greater social withdrawal and hyperactivity (r = −0.556, *p* = 0.048 and r = −0.560, *p* = 0.049, respectively). **Conclusions:** The present cohort study of children with SMS studied in real-life conditions, using both objective and subjective measures, shows that patients continued to experience sleep and behavioral disturbances despite treatment. Exploratory analyses identified distinct associations between subjective sleep disturbances, circadian rhythm characteristics, and specific behavioral domains. While subjective sleep disturbances were associated with irritability, stereotypic behavior, and inappropriate speech, circadian rhythm parameters are specifically associated with hyperactivity. Both were also associated with social withdrawal. As these findings arise from an exploratory observational secondary analysis, they should be considered hypothesis-generating and require confirmation in larger prospective studies. If confirmed, they may help identify sleep and circadian rhythm characteristics as potential targets for future interventions in children with SMS.

## 1. Introduction

Smith–Magenis syndrome (SMS) (OMIM #182290) is a rare neurodevelopmental disorder characterized by a combination of cognitive impairment, severe behavioral disturbances, and sleep disorders [1,2]. The estimated prevalence ranges from 1 in 15,000 [3] to 1 in 25,000 individuals [4]. In approximately 90% of cases, SMS is caused by a microdeletion on chromosome 17p11.2; less frequently, it results from a pathogenic variant in the *RAI1* gene, located within the same region [5,6].

Sleep disturbances are a core feature of SMS and often serve as a key diagnostic clue. These disturbances typically include difficulties maintaining sleep, early morning awakenings, reduced total sleep time, and excessive daytime sleepiness [7]. Normally, sleep is regulated by a sophisticated interaction between a homeostatic and a circadian process, involving a precisely timed circadian rhythm of melatonin secretion, which peaks at night and is suppressed during the day by light exposure [8,9]. In SMS, this rhythm was described to be inverted, with a melatonin peak occurring around midday [10,11,12,13]. Objective sleep assessments are coherent with this observation, revealing a shift in the midpoint of sleep consistent with an advanced circadian phase [7,13].

This circadian misalignment may directly contribute to poor sleep quality and increased nighttime alertness, as well as excessive daytime sleepiness [10,11]. Beyond sleep, melatonin abnormalities may also influence the marked behavioral symptoms seen in SMS—such as aggression, self-injury, and stereotypies—as previously proposed [14,15]. Similar associations between sleep problems and behavioral outcomes have been reported in children without neurogenetic disorders [16,17]. Consequently, it is recommended that sleep disturbances in children with SMS be systematically screened and treated—notably with prolonged-release melatonin supplementation in the evening, with or without beta-blockers in the morning—in conjunction with standard behavioral and cognitive interventions.

SMS shares several characteristics with Autism Spectrum Disorder (ASD), including certain behavioral features and comorbidities. Sleep disorders are also very common in ASD, affecting 50% to 80% of children. Recently, Yavuz-Kodat and colleagues showed that, in a group of 52 children with ASD aged 3 to 10 years, monitored using actigraphy, shorter periods of continuous sleep were associated with irritability and stereotypical behaviors. In these children, however, circadian characteristics were less prominently involved in behavioral disturbances [18]. Thus, it is essential to distinguish between sleep- and circadian-related difficulties as these may differentially impact daytime functioning and may require distinct therapeutic approaches.

Although sleep and circadian disturbances are well documented in SMS, their respective contributions to the behavioral phenotype remain poorly understood. In our previous study, we characterized sleep architecture, circadian rest–activity rhythms, and the effects of sleep-targeted treatments in children with SMS, demonstrating persistent abnormalities despite widespread use of prolonged-release melatonin and, in some cases, beta-blockers [13]. However, the relationships between objective and subjective measures of sleep and circadian rhythms and specific behavioral domains were not investigated. The present secondary analysis therefore aimed to examine whether sleep continuity and circadian rhythm characteristics, assessed using questionnaires and home-based actigraphy, are differentially associated with behavioral difficulties in children with SMS. We hypothesized that markers of sleep fragmentation and circadian phase and rhythm robustness would show distinct, domain-specific associations with behavioral outcomes.

## 2. Materials and Methods

### 2.1. Patients and Study Design

The present study is a secondary analysis of a previously published single-center cohort described in Comajuan et al. [13]. The original study prospectively enrolled 20 children with SMS, aged 5 to 13 years, followed in the pediatric unit of the Hôpital Psychiatrique du Vinatier (GenoPsy Team, Lyon, France), who underwent a sleep assessment at the sleep unit of the Hôpital Femme Mère Enfant (Hospices Civils de Lyon, Lyon, France) between 2022 and 2024. The study was conducted in accordance with Good Clinical Practice guidelines and the Declaration of Helsinki (1975, revised in 2008). Approval was granted by the Sud-Est II Ethics Committee (19 October 2021, EudraCT number 2020-A03279-30). Written informed consent was obtained from both parents of participants, and assent was obtained from the children whenever possible.

**Figure 1:**
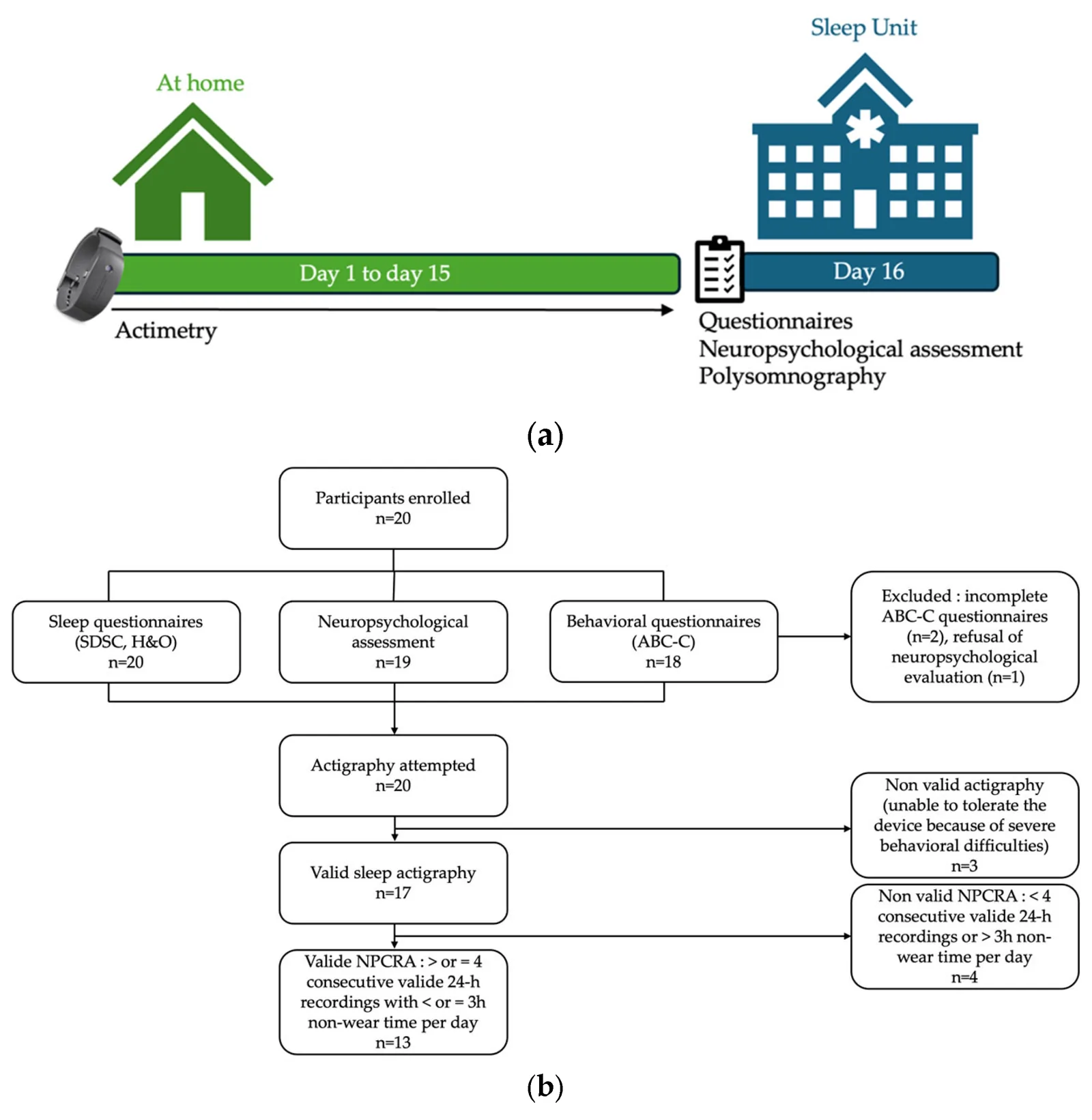
(**a**) Study design. (**b**) Participant flow. Flow diagram of participant inclusion and data availability. All 20 participants completed the sleep questionnaires (SDSC and H&O). Two ABC-C questionaries were excluded because they were incomplete or not interpretable. Three children were unable to tolerate the actigraph owing to severe behavioral difficulties, preventing sleep actigraphy analyses. Non-parametric circadian rhythm analysis (NPCRA) additionally required at least four consecutive valid 24 h recordings with ≤3 h of non-wear time per day, resulting in circadian measures being available for 13 participants. Consequently, the sample size varied across correlation analyses according to data availability.

Children who agreed to take part in the study were provided with an actigraphy device, which they wore at home for 15 days, without any change to their usual treatment plan, before attending a polysomnographic (PSG) recording at the sleep unit. During hospitalization, children underwent neuropsychological assessment, completed sleep and behavioral questionnaires, and underwent polysomnographic assessment under controlled light conditions. Light exposure was maintained below 20 lux throughout the hospitalization period. The study timeline and participant flow are summarized in Figure 1.

### 2.2. Data Collection

The age, sex, height, weight, and PSG data for each child were obtained during their hospitalization in the sleep unit. Age at diagnosis, genetic testing results, comorbidities (intellectual developmental disorder [IDD], ASD), and treatments (date of introduction, dosage) were collected from their medical files.

### 2.3. Sleep and Circadian Measures

#### 2.3.1. Sleep–Wake Quality

Sleep quality was assessed for each child both subjectively, using the Sleep Disturbance Scale for Children (SDSC) questionnaire, and objectively using actigraphy.

The SDSC is a frequently used screening scale for sleep disorders in children, completed by parents, validated in French and adapted to age (version 4 to 16 years). A score >45/125 was considered pathological. The score was also used to specify the type of disorder encountered according to specific sub-domains: insomnia (>21/35), parasomnia (>17/21), sleep breathing disorders (>12/25), non-restorative sleep (>11/15), and excessive daytime sleepiness (>5/15) [19,20].

To objectively measure sleep–wake quality, actigraphy recordings were conducted in real-life conditions, under stable treatment. Each child was asked to wear an activity monitor (ActiWatch^®^, Philips Respironics, Murrysville, PA, USA, CE certification no. 1104768) for 15 days at home. Actigraphs were attached to the non-dominant wrist of the children and removed only during times when it could get wet (e.g., shower). Children were monitored during the academic year, including both weekdays and weekends. During this 15-day period, parents also completed a sleep diary on each study day, documenting bedtime, wake-up time, daytime naps, and any periods during which the actigraph was removed. Non-wear periods were identified from the sleep diary and systematically confirmed by visual inspection of the actigraphy recordings by a trained investigator. Data for each actigraph measure were aggregated (averaged) separately for weekday (scheduled, SC) and weekend (free, FR) nights, which were used as the units for analysis. For descriptive analyses, weekday and weekend values were reported separately when relevant (e.g., sleep midpoint). For correlation analyses with behavioral outcomes, participant-level mean actigraphy measures across all valid recording days were used. Daytime naps were analyzed separately and were not included in the calculation of nocturnal sleep variables. Sleep diaries were used to verify bedtime and wake-up time and to identify periods during which the device had been removed. Data were analyzed in 30 s epochs and translated into sleep measures by the software Actiware (version 6.1.2, Philips), as previously described [13,21]. Data were evaluated at a medium-sensitivity threshold. Actigraphic sleep measures for the analysis included the following: bedtime (as indicated in the diary), sleep start time (defined as the first minute of at least three consecutive minutes of scored sleep), sleep end time (defined as the last minute of at least five consecutive minutes of scored sleep just prior to the wake up time reported in the sleep diary), time spent in bed (TSB, defined as the difference between bedtime and wake up time), sleep period (SP, defined as the difference between sleep start time and sleep end time), sleep latency (SL, defined as the difference between bedtime and sleep start time), total sleep time (TST, number of minutes scored as sleep in the SP, pathological values < 8 h), fragmentation index (normal values: ≤30/h), sleep efficacy (TST/TSB), sleep efficiency (TST/SP*100; pathological values ≤ 85%), wake after sleep onset (WASO, wake time after falling asleep, pathological values ≥ 60 min), existence of napping times, and length of naps. Normative values for age were obtained from Galland et al. [22].

In addition to the classical sleep measures, we determined the longest continuous sleep episode (LSE) for each child [18]. The LSE is defined as the longest duration of uninterrupted sleep, or sleep interrupted by less than five consecutive minutes of wakefulness, as recorded by actigraphy.

#### 2.3.2. Circadian Measures

The Horne and Östberg Morningness–Eveningness Questionnaire (H&O) is composed of five questions to assess an individual’s chronotype (2 to 18 years): definitely of evening type (score 5–8), moderately of evening type (9–11), intermediate type (12–17), moderately of morning type (18–20), and definitely of morning type (21–25). The questionnaire was completed by the parents, with the help of the child when possible.

Non-parametric circadian rhythm analyses (NPCRA) were also performed. Algorithms that determine these variables were described by Van Someren et al. [23]. To perform NPCRA, at least four 24 h days of monitoring were required (including data regarding potential daytime naps), whereas only nighttime recordings of at least 4 days were required for sleep analysis. If the actigraph device was removed for more than half an hour and up to 3 h a day, the activity level of this missing period was replaced by the average of the mean activity level of the day before and the day after this missing period. If more than 3 h of recording was missing in a day, that period was excluded. NPCRA included the following parameters: L5, M10, interdaily stability (IS), intradaily variability (IV), and relative amplitude (RA). L5 corresponds to the 5 h period with the least movements (where the sum of the movement index is minimal), a proxy of the deepest sleep over the 24 h period, and the L5 onset to the clock time which is the starting point of the L5 period. The M10 corresponds to the 10 h period with the highest amount of movement (where the sum of the movement index is maximal), and the M10 onset to the clock time which is the starting point of the M10 period. The L5 and M10 onset times are indicators of circadian phase. IS gives an indication on the day-to-day stability of the rest–activity rhythm. IS values range between 0, for no stability, to 1, indicating that the rest–activity pattern is repeated perfectly every single day. A higher IS value indicates a more stable rhythm. IV provides information on the variability of the rhythm within a day and quantifies the degree of fragmentation of the behavioral rhythm. In other words, IV indicates the frequency and extent of transitions between rest and activity. Its value ranges between 0, indicating that transitions between rest and activity within a day are tightly consolidated, and 2, which means that the fragmentation of transitions between rest and activity is random. RA is an estimate of the strength or robustness of the circadian rhythm. It is defined as the normalized difference between the most active 10 h period and least active 5 h period in a 24 h pattern. Higher values indicate greater amplitude with values ranging between 0 and 1. The normative values in children (mean 11.6 years) are defined as follows: IS 0.55 ± 0.13, IV 0.76 ± 0.20, M10 midpoint 15h17 ± 1h29 (10h17 ± 1h29 start time), L5 midpoint 03h21 ± 1h21 (00h59 ± 1h21 start time), and RA 0.87 ± 0.07 [24].

The midpoint of sleep was calculated using data from actigraphic recordings. This measure is indicative of the circadian preference of a child, i.e., its chronotype. Bedtime, SL, and wake-up time during weekdays and weekend days were used to calculate sleep onset and sleep duration. Then, the midpoint of sleep was calculated on weekdays (MSF) and weekend days (MSW) according to the Munich questionnaire method [25]. Normative data for the midpoint of sleep were obtained from unpublished data (provided by a co-author [SP]) from the Elfe cohort study [26], which were calculated by including 5 weekdays and 2 weekend days. These varied according to age as follows: 2.1 ± 0.4 in children aged 5.5 years old and 2.3 ± 0.4 in children aged 10.5 years.

### 2.4. Cognitive and Behavioral Difficulties

The neuropsychological assessment was carried out by a single neuropsychologist (M.N.B) expert in SMS, under the usual test-taking conditions.

The behavioral profile of the children was analyzed using the Aberrant Behavior Checklist-Community (ABC-C) rating scale [27], which is particularly adapted to people with IDD. It consists of 58 items completed by parents and is divided into five subscales, each focusing on different behavioral domains: (I) Irritability, Agitation, Crying; (II) Lethargy, Social Withdrawal; (III) Stereotypic Behavior; (IV) Hyperactivity, Noncompliance; and (V) Inappropriate Speech. Each item is rated based on the frequency of the behavior in the past few weeks. The items are scored on a 4-point Likert scale (0 = Not a problem at all, 1 = Slight problem, 2 = Moderate problem, 3 = Severe problem). The total score for each subscale is the sum of the individual item scores within that subscale. Higher scores indicate greater severity or frequency of problematic behaviors.

The 48-item Conners’ Parent Rating Scale (CPRS-48) [28] was used to assess behavioral and attention disorders. This scale is divided into six components (conduct, learning, psychosomatic, impulsivity, anxiety, and hyperactivity), the scores of which are standardized according to age groups. Moderate to severe symptoms are defined with a cut-off above 65, and severe symptoms are defined with a cut-off above 75.

The Vineland Adaptive Behavior Scales (VABS) is a 117-question test for parents, measuring adaptive skills in 4 domains (communication, daily living skills, socialization, and motor skills), each divided into 11 sub-domains, which enable us to obtain equivalent ages for each domain.

The age-dependent Wechsler scales (WPPSI IV for children aged from 2 years and 6 months to 7 years and 3 months; WISC V for children aged from 6 years to 16 years and 11 months) were used to calculate intellectual efficiency. Five domains were analyzed to study reasoning abilities: verbal comprehension index (VCI), visuo-spatial index (VSI), fluid reasoning index (FRI), working memory index (WMI), and processing speed index (PSI). A total intelligence quotient (IQ) was calculated from the results obtained in each test; normative values range from 85 to 115. The degree of IDD was determined by the neuropsychologist who administered all the scales. IDD is defined by the DSM-5 as a neurodevelopmental disorder that appears during the developmental period and is characterized by limitations in both intellectual functioning (IQ < 70) and adaptive capacities. The Vineland and Wechsler scales are therefore highly complementary, and their combined use provides a good-quality cognitive profile.

### 2.5. Statistical Analyses

An initial descriptive table was created to better characterize the population, including the presence of comorbid conditions (IDD, ASD, or ADHD), and the results of the Vineland, ABC-C, SDSC, H&O and actigraphy assessments. Subgroup analyses are presented based on whether or not patients were taking beta-blockers and psychostimulants. Due to the small number of patients, only comparisons between patients taking and those not taking psychostimulants were performed, using Mann–Whitney tests for continuous variables and Fisher’s exact test for categorical variables.

Correlations between subjective and objective sleep circadian variables and the five subscales of the ABC-C were estimated using Spearman’s correlation test. As measures were not always interpretable (e.g., incomplete questionnaires, insufficient number of days of actigraphy, etc.), the number of participants for which the measure was available is specified for each analysis. Due to the small number of patients in each treatment group, it was not possible to perform adjusted analyses based on treatment. The correlation analyses were exploratory. Given the exploratory nature of this secondary analysis and the limited sample size, no adjustment for multiple comparisons was performed. Therefore, reported *p*-values should be considered nominal and interpreted as hypothesis-generating.

Statistical analysis was performed using Jamovi version 2.6.2. Statistical significance was set at *p* < 0.05.

## 3. Results

### 3.1. Children’s Characteristics

Among the 20 children included, 11 (55%) were female, and the mean age was 10 years (range: 5 years and 5 months to 13 years and 8 months). Overall, 15 (75%) children were carriers of a chromosome 17p11.2 microdeletion and 5 (25%) had an *RAI1* gene mutation. Three patients were obese, four were overweight, and one was moderately underweight. The PSG data showed that obstructive sleep apnea was present in 10/19 (53%) children, including 2 severe cases with an obstructive apnea hypopnea index of 17.6 and 29.7 per hour. These two subjects were adolescents with obesity. Among the 19 children with available complete neuropsychological assessment, 10 (53%) had no IDD, 7 (37%) had moderate IDD, and 2 (10%) had mild IDD. Overall, 5/20 (25%) children had an associated ASD (Table 1). Regarding treatments, 19/20 (95%) children were on prolonged-release melatonin (1 to 20 mg daily) and 4/20 (20%) were on beta-blockers (0.8 to 8.7 mg/kg/d). Among other prescribed treatments, 11/20 (55%) children were receiving psychostimulants treatment (9/11 with extended-release or modified-release methylphenidate), 2/20 (10%) on antipsychotics (risperidone), and 2/20 (10%) on neuroleptic antihistamines (alimemazine). One (5%) patient was also taking an antidepressant (sertraline).

### 3.2. Sleep and Circadian Measures

The availability of neuropsychological, behavioral, sleep, and circadian data for each analysis is summarized in Figure 1b. According to the SDSC results, pathological sleep disturbances were present in 14/20 (70%) children and concerned mainly complaints of insomnia (10/20, 50%) and excessive daytime sleepiness (8/20, 40%). Overall, 11 (55%) children reported taking naps, lasting an average of 33 min per day (ranging from 4 min to 1 h and 21 min): 6 (30%) reported taking naps during the week, lasting an average of 50 min (ranging from 20 to 90 min), and 10 (50%) during weekends, lasting an average of 53 min (range 15 to 120 min). Five patients took daytime naps on both weekdays and weekends. The Horne and Ostberg chronotypes confirmed the morning type in 70% of patients (moderately and definitely, 14/20, 10 of whom had a score > 21) and an intermediate type in the remaining 30% (6/20), and no patient had an evening chronotype.

Actigraphy sleep data were available for 17 patients, as 3 patients did not tolerate wearing the device due to significant behavioral difficulties. A total of 151 nights were recorded, corresponding to a mean of 8.9 nights per child (SD = 4.1, range: 2–14). Actigraphy revealed reduced TST in 12/17 (71%) children. Reduced sleep efficiency was observed for 15/17 (88%) children with long nighttime awakenings (WASO >60 min in 10/17, 59% but with little fragmentation (>30/h in 2/17, 12%). The mean sleep start time was 21:30 (20:20–22:39) with SL > 30 min in only 3/17 (18%) children. Circadian measures obtained by actigraphy were available in 13 children, because NPCRA required at least four consecutive valid 24 h recordings with no more than 3 h of non-wear time per day, a criterion that was not met in four additional participants. An early L5 onset (<23:38) was found in 12/13 (92%) children and an early M10 onset (<09h12) in 8/13 (62%) children. While the mean IS, IV, and RA were in normal ranges, high IS and RA were found in respectively 6/13 (46%) and 9/13 (69%) children. The mean MSF and MSW were similar at 01:31 (00:46–02:26) and 01:13 (23:46–02:11), respectively, and tended to occur earlier than in healthy children (Table 1).

**Table 1:** A. Patient characteristics. B. Actigraphy characteristics. Characteristics of the 20 children with Smith–Magenis Syndrome. Actigraphic variables are presented as mean (range). For sleep and circadian variables with published reference values, the indented rows indicate the number of participants outside the reference range (n/N).

	Total Cohort N = 20	Beta Blockers No (N = 16) Yes (N = 4)		Psychostimulants No (N = 9) Yes (N = 11)	
**A. Patient characteristics**					
**Clinical characteristics**					
**Age, years, mean (SD)**	**10.0 (2.5)**	10.0 (2.6)	10.4 (2.6)	9.2 (2.4)	10.8 (2.5)
**Sex, female, n (%)**	11 (55)	2 (13)	2 (50)	5 (55)	6 (55)
**Comorbidities, n (%)**					
IDD (n = 19)	9 (47)	2 (13)	2 (50)	7 (78)	3 (27)
ASD (n = 20)	5 (25)	2 (13)	2 (50)	4 (44)	1 (10)
ADHD (n = 20)	15 (75)	2 (513)	2 (50)	**4 (44)** *	**11 (100)** *
**Questionnaires**					
**VABS subdomains, developmental age in years, mean (range), (n = 15)**					
Communication	4.0 (1.3–7.3)	3.9 (1.7–6.1)	4.3 (1.3–7.3)	**2.6 (1.3–4.4)** **	**5.3 (3.8–7.3)** **
Daily living skills	4.5 (1.1–8.8)	4.5 (1.1–7.4)	4.7 (1.1–8.8)	**2.8 (1.1–5.3)** **	**6.0 (3.3–8.8)** **
Socialization	3.4 (1.0–7.3)	3.6 (1.0–7.3)	2.7 (1.3–4.6)	**2.0 (1.0–3.8)** **	**4.6 (3.1–7.3)** **
**ABC-C subscales, mean (range), (n = 18)**					
I-Irritability, agitation, crying	46.9 (17.8–82.2)	46.5 (17.8–82.2)	48.9 (33.3–68.9)	50.6 (22.0–82.2)	44.1 (17.8–69.0)
II-Lethargy, social withdrawal	15.3 (0.0–56.3)	14.9 (0.0–56.3)	17.4 (0.0–45.8)	17.4 (4.0–56.3)	13.4 (0.0–46.0)
III-Stereotypic behavior	24.9 (0.0–76.2)	26.3 (0.0–76.2)	17.5 (0.0–42.9)	22.1 (0.0–76.2)	27.2 (0.0–52.0)
IV-Hyperactivity, noncompliance	52.1 (2.1–97.9)	55.3 (2.1–97.9)	36.1 (18.8–45.8)	51.1 (2.0–93.8)	52.0 (27.0–97.9)
V-Inappropriate Speech	38.4 (0.0–100.0)	40.0 (0.0–100.0)	30.6 (8.3–50.0)	29.2 (0.0–58.3)	45.8 (8.3–100.0)
Total score	30.5 (0.0–66.7)	37.5 (9.0–66.7)	31.7 (20.1–51.0)	36.8 (9.0–66.7)	36.3 (14.9–62.6)
**SDSC, mean (range), (n = 20)**					
Insomnia (/35)	18.1 (11–31)	18.5 (11–31)	16.5 (11–20)	18.1 (11–31)	18.1 (11–23)
Parasomnia (/35)	11.0 (7–18)	11.3 (7–18)	9.5 (7–11)	11.2 (7–18)	10.7 (7–15)
Sleep breathing disorders (/25)	7.5 (5–12)	7.3 (5–12)	8 (6–12)	6.9 (5–12)	7.9 (6–12)
Non-restorative sleep (/15)	4.2 (2–7)	4.3 (2–7)	3.8 (2–6)	3.9 (2–6)	4.4 (3–7)
Excessive daytime sleepiness (/15)	5.8 (3–8)	5.7 (3–8)	6.0 (3–8)	6.3 (4–8)	5.3 (3–7)
Total score (/125)	46.6 (31–57)	47.3 (31–57)	43.8 (39–48)	46.7 (31–55)	46.5 (38–57)
**H&O, mean (range)**	20.0 (13.0–25.0)	20.5 (13.0–25.0)	17.5 (14.0–21.0)	19.7 (15.0–24.0)	20.1 (13.0–25.0)
**B. Actigraphic characteristics**					
**Sleep measures, mean (range), (n = 17)**					
Sleep start time (hh:mm)	21:30 (20:20–22:39)	21:30 (20:48–22:30)	21:36 (20:18–22:36)	21:30 (20:18–22:30)	21:30 (20:48–22:36)
Sleep end time (hh:mm)	06:19 (04:14–07:53)	06:13 (04:12–07:54)	06:24 (05:06–07:42)	06:42 (05:00–07:54)	06:00 (04:12–07:06)
TST (min)	457.2 (336.3–456.2)	454 (336–543)	462 (344–568)	471 (421–568)	446 (336–543)
Below reference (<480 min), n/N	12/17	2/4	10/13	5/7	7/10
WASO (min)	71.1 (4.8–250.0)	75.7 (33.5–193.0)	60.3 (17.8–111.0)	87.1 (17.8–193.0)	61.5 (33.5–100.0)
Above reference (≥60 min), n/N	10/17	2/4	8/13		
SE (%)	77.9 (40.0–96.7)	78.5 (62.7–88.3)	76.3 (68.6–81.6)	74.3 (62.7–81.6)	80.6 (75.0–88.3)
Below reference (<85%), n/N	15/17	4/4	11/13	7/7	8/10
SL (min)	22.9 (0.0–137.8)	19.0 (4.8–32.4)	34.3 (17.6–74.4)	29.0 (11.6–74.4)	18.1 (4.8–32.4)
Above reference (>30 min), n/N	3/17	1/4	2/13	2/7	1/10
Fragmentation (%)	23.7 (10.0–48.0)	24.7 (15.2–37.5)	19.9 (9.2–30.9)	24.3 (9.2–37.5)	23.1 (15.2–30.9)
Above reference (≥30/h), n/N	2/17	1/4	1/13	1/7	1/10
Number of wake episodes	38.6 (8.0–145.0)	37.5 (27.6–53.8)	38.8 (19.0–68.6)	37.4 (19.0–68.9)	38.1 (23.9–53.8)
Mean wake episode (min)	2.0 (0.3–11.9)	2.3 (1.1–7.7)	1.5 (0.8–1.9)	2.6 (0.9–7.6)	1.7 (1.1–4.1)
LSE (min)	419 (301–609)	404 (301–531)	471 (365–609)	441 (301–609)	405 (309–522)
**Circadian measures, mean (range), (n = 13)**					
IS	0.65 (0.46–0.79)	0.65 (0.46–0.79)	0.66 (0.60–0.75)	0.61 (0.46–0.73)	0.70 (0.63–0.79)
Below reference (<0.42), n/N Above reference (>0.68), n/N	0/13 6/13	0/3 1/3	0/3 5/10	0/6 2/6	0/7 4/7
IV	0.60 (0.48–0.97)	0.56 (0.48–0.63)	0.75 (0.58–0.97)	0.62 (0.51–0.97)	0.59 (0.48–0.71)
Below reference (<0.56), n/N Above reference (>0.96), n/N	5/13 1/13	5/10 0/6	0/3 1/3	3/6 1/6	2/7 0/7
RA	0.94 (0.90–0.98)	0.95 (0.90–0.98)	0.94 (0.91–0.96)	0.95 (0.90–0.98)	0.95 (0.91–0.98)
Below reference (<0.80), n/N Above reference (>0.94), n/N	0/13 9/13	0/10 7/10	0/3 2/3	0/6 4/6	0/7 5/7
L5-onset (hh:mm)	22:30 (19:54–23:42)	22:18 (19:54–23:42)	23:06 (22:54–23:12)	22:42 (21:36–23:42)	22:18 (19:54–23:24)
Below reference (<23:38), n/N Above reference (>02:20), n/N	12/13 0/13	9/10 0/10	3/3 0/3	5/6 0/6	7/7 0/7
M10-onset (hh:mm)	09:17 (08:19–10:24)	09:12 (08:19–10:24)	09:32 (09:06–09:57)	**09:44 (09:01–10:24)** *	**08:53 (08:19–09:57)** *
Below reference (<09:12), n/N Above reference (>11:49), n/N	8/13 0/13	7/10 0/10	1/3 0/3	2/6 0/6	6/7 0/7
MSW (hh:mm)	01:13 (23:46–02:11)	01:06 (23:18–02:12)	01:36 (01:18–01:48)	01:24 (00:54–02:12)	01:06 (23:18–01:54)
MSF (hh:mm)	01:31 (00:46–02:26)	01:36 (23:48–02:24)	01:24 (00:48–01:48)	01:42 (00:48–02:24)	01:24 (23:48–02:12)

SD, Standard Deviation; IDD, intellectual developmental disorder; ASD, Autism Spectrum Disorder; ADHD, Attention deficit hyperactivity disorder; VABS, Vineland Adaptive Behavior Scales; ABC-C, Aberrant Behavior Checklist-Community; SDSC, Sleep Disturbance Scale for Children; TST, Total Sleep Time; WASO, Wake After Sleep Onset; SE, Sleep Efficiency; SL, Sleep Latency; LSE, Longest Sleep Episode; IS, Interdaily Stability; IV, Intradaily Variability; RA, Relative Amplitude; L5-onset, start time of the 5 h period with the least movements; M10-onset, start time of the 10 h period with the highest amount of movement; MSW Midpoint of Sleep on weekend days; MSF, Midpoint of Sleep on weekdays. * *p* < 0.05; ** *p* < 0.01 using non-parametric Wilcoxon–Mann–Whitney test (if continuous variables) or Fisher’s exact test (if binary categorical variables).

### 3.3. Behavioral Measures

According to the answers reported by parents on the ABC-C scale, the behavioral domains with the highest mean scores were hyperactivity and noncompliance (mean 52.1, range 2.1–97.9), and irritability, agitation, and crying (mean 46.9, range 17.8–82.2; Table 1). Results of the CPRS-48 showed the presence of learning problems in 18/20 (90%) patients, which were severe in 13/18 (72%). Hyperactivity was present in 16/20 (80%) patients, impulsivity in 10/20 (50%), and anxiety in 4/20 (20%); psychosomatic problems were less common (3/20, 15%).

In the analysis by treatment group, children on psychostimulants were more likely to have ADHD (100% vs. 44%, *p* < 0.01), better scores in the areas of communication (5.3 vs. 2.6, *p* < 0.01), daily living skills (6.0 vs. 2.8, *p* < 0.01), and socialization (4.6 vs. 2.0, *p* < 0.01), and an earlier M10 start time (08:53 vs. 09:44, *p* = 0.02) (Table 1). No differences were found for the ABC-C scales and sleep-activity measures of actimetry.

### 3.4. Correlations Between Measures of Sleep and Circadian Rhythm and Behavioral Difficulties

We then examined correlations between subjective and actimetry-derived objective sleep and circadian measures and behavioral difficulties across the five ABC-C domains (Table 2).

Sleep disorder scores were positively correlated with stereotyped behaviors (r = 0.472, *p* = 0.048). Insomnia scores were positively correlated with irritability, social withdrawal, and stereotyped behaviors (r = 0.472, *p* = 0.048; r = 0.701, *p* = 0.001; and r = 0.648, *p* = 0.004, respectively). Non-restorative sleep scores were positively correlated with inappropriate speech (r = 0.476, *p* = 0.046).

In contrast, there was no significant association between objective sleep measures and behavior in this cohort.

Interestingly, whereas subjective measures indicated that children with a more pronounced morning chronotype were less irritable (r = −0.565, *p* = 0.015), objective actigraphy measures showed that an earlier timing of the least active 5 h period (L5 onset) was associated with increased social withdrawal (r = −0.556, *p* = 0.048) and hyperactivity (r = −0.560, *p* = 0.049).

## 4. Discussion

The present work represents a secondary analysis of our previously reported SMS cohort, specifically focusing on the relationship between sleep/circadian measures and behavioral outcomes, which had not been addressed in the original report. This study, conducted among children with SMS in real-life conditions, using both objective and subjective measures, shows that patients continued to experience behavioral and sleep disturbances despite treatment [13]. Exploratory analyses identified distinct associations between subjective sleep disturbances, circadian rhythm characteristics, and specific behavioral domains. While subjective sleep disturbances were associated with irritability, stereotypic behavior, and inappropriate speech, circadian rhythm measures were specifically associated with hyperactivity. Both were also associated with social withdrawal.

### 4.1. Behavioral Difficulties and Sleep Disturbances: Objective and Subjective Data

The impact of sleep disorders on neurodevelopment is well established. In the present cohort, children with behavioral difficulties related to irritability, social withdrawal and stereotypic behavior had significantly more insomnia complaints. Behavioral difficulties related to stereotypic behavior were associated with greater sleep disorder complaints, and inappropriate speech was associated with non-restorative sleep. These findings suggest an association between subjective sleep disturbances and behavioral difficulties in children with SMS, as previously reported by Poisson et al. [5]. These findings are consistent with the broader literature indicating that sleep continuity is a key determinant of daytime socio-emotional functioning in neurodevelopmental disorders [29]. The absence of significant correlations between objective sleep data, as measured by actigraphy, and behavioral outcome may have several explanations. First, the limited sample size reduced the statistical power of these analyses. Second, subjective and objective sleep measures capture partially distinct dimensions of sleep disturbance. Parent-reported insomnia may reflect clinically relevant sleep difficulties that are not fully captured by actigraphy, including difficulties at bedtime, nighttime behaviors, and caregiver perception of daytime consequences. Conversely, wrist actigraphy has limited sensitivity for detecting brief arousals and may misclassify quiet wakefulness as sleep, particularly in children with neurodevelopmental disorders [29]. Therefore, the absence of associations with actigraphy parameters does not exclude a clinically meaningful relationship between sleep disruption and behavioral difficulties in SMS. It should be noted that, in children with SMS presenting with ADHD symptoms, psychostimulant treatment may have a positive impact on adaptive functioning. In our cohort, children receiving psychostimulants showed better communication, socialization, and daily living skills scores than those not receiving such treatment, although the observational design of the study does not allow conclusions regarding causality. They also exhibited an earlier M10 onset, possibly reflecting increased daytime activity or alertness rather than a true shift in circadian phase, as no differences were observed in other circadian rhythm parameters, particularly in L5 onset.

### 4.2. Behavioral Difficulties and Circadian Rhythm Parameters

In the present cohort, social withdrawal and hyperactivity were associated with an earlier onset of L5, indicating that a greater degree of circadian phase advance was associated with more severe behavioral difficulties. These findings are consistent with the well-described circadian dysfunction observed in SMS and suggest that circadian rhythm alteration may be linked to daytime behavioral difficulties. However, given the exploratory nature of this observational secondary analysis, these associations should be interpreted cautiously and confirmed in larger prospective studies. Interestingly, although this may appear paradoxical, subjective chronotype assessment using the Horne–Östberg questionnaire indicated that children were less irritable when their sleep–wake behavior was aligned with their usual circadian preference. This discrepancy suggests that subjective chronotype and objective markers of circadian timing may capture different dimensions of circadian functioning. On the other hand, the sleep midpoint may not be the most appropriate marker of circadian timing in SMS, as prolonged nocturnal awakenings, which are common in this population, are not taken into account in its calculation, whereas they are reflected in the L5-onset measure. Consequently, L5 onset may represent a more sensitive indicator of circadian abnormalities and their behavioral consequences in SMS. No significant associations were found between the other circadian parameters examined and the behavioral domains assessed, which may reflect the limited sample size and the considerable inter-individual variability characteristic of this disorder.

Among the four children taking beta-blockers in the initial polysomnographic study (on beta-blockers without exogenous melatonin), bedtimes and wake-up times tended to be later, with a delayed sleep onset and more fragmented sleep, which may be associated with poorer sleep efficiency [13]. Inhibition of endogenous daytime secretion could therefore be associated with a phase delay.

The small number of patients on beta-blockers did not allow for statistical analyses in this specific subgroup, particularly to determine whether a shift in circadian timing toward a delayed phase had a beneficial effect on various aspects of the behavioral disturbances associated with this syndrome.

### 4.3. Study Limitations

The present findings should nevertheless be interpreted in light of several limitations.

First, the relatively small sample size reduced the statistical power to detect associations, particularly for the objective actigraphy-derived measures.

Next, actigraphy sleep data were not available for three children because they were unable to tolerate wearing the device owing to severe behavioral difficulties. In addition, NPCRA could only be performed in participants with at least four consecutive valid 24 h recordings, each containing no more than 3 h of non-wear time, resulting in a further reduction in sample size for circadian analyses. This missingness may have introduced selection bias, as children with the most severe behavioral phenotype may have been underrepresented in the objective sleep and circadian analyses. Consequently, the observed associations may underestimate the true relationship between sleep, circadian disturbances, and behavioral difficulties in SMS.

Another important limitation is the potential for residual confounding. Nearly all participants were receiving prolonged-release melatonin, more than half were treated with psychostimulants, and a smaller proportion received beta-blockers or other psychotropic medications. In addition, obstructive sleep apnea was present in approximately half of the cohort. Each of these factors may independently influence sleep architecture, circadian rest–activity rhythms, and behavioral functioning. Although exploratory subgroup analyses were performed for psychostimulant treatment, the limited sample size precluded multivariable adjusted analyses. It is therefore difficult to determine whether the observed phase advance reflects the intrinsic circadian phenotype of SMS or treatment-related effects. Consequently, the observed associations should be interpreted cautiously and considered hypothesis-generating rather than evidence of independent effects.

Another limitation is that circadian phase was inferred from actigraphy-derived rest–activity parameters rather than from physiological markers of the endogenous circadian clock. Although L5 and M10 timing provide valuable information on behavioral circadian organization in real-life conditions, they cannot replace direct measures such as dim-light melatonin onset (DLMO). Therefore, our findings should be interpreted as reflecting alterations in behavioral circadian timing rather than definitive evidence of intrinsic circadian phase abnormalities. At the same time, we believe it is important to interpret these limitations in the context of Smith–Magenis syndrome, a rare neurodevelopmental disorder with an estimated prevalence of approximately 1 in 15,000/25,000 individuals. Recruiting a sufficiently large and clinically homogeneous cohort remains inherently challenging. To our knowledge, no previous study has combined such a comprehensive assessment of sleep and circadian rhythms in this population, integrating both polysomnography and prolonged actigraphy with detailed behavioral phenotyping. Furthermore, our study was conducted under real-life conditions, providing clinically relevant information that may help guide the management of sleep disturbances in everyday practice. Beyond advancing the understanding of sleep and circadian alterations in SMS, these findings may help guide future research and generate hypotheses regarding the potential effects of commonly prescribed medications, particularly psychostimulants, in this population. Future studies with larger cohorts, and longitudinal designs, will be essential to further disentangle these complex relationships. An analysis of the effects of sleep on behavior in untreated children could also clarify the contribution of circadian dysfunction to behavioral manifestations in this population.

### 4.4. Comparison with Findings in Autism Spectrum Disorder

The findings of the present study are broadly consistent with those reported by Yavuz-Kodat et al. in children and adolescents with ASD [18]. The authors found associations between sleep quality, notably LSE, and irritability and stereotypical behaviors. Similarly, we found associations between sleep disturbances and certain behavioral domains. However, these associations involved different sleep parameters, with circadian rhythm measures, particularly L5 onset, being associated with social withdrawal and hyperactivity. In contrast, no significant association was observed between behavioral difficulties and LSE in our cohort. These differences may be partly explained by the smaller sample size, older age, and lower proportion of individuals with intellectual developmental disability (IDD) in the present cohort, as well as by the distinct pathophysiological characteristics of SMS and ASD. Indeed, whereas the most common sleep complaints in ASD include difficulties initiating and maintaining sleep, often accompanied by a delayed sleep phase [14,17,18], SMS is characterized by marked circadian dysregulation, including shortened sleep latency and early morning awakenings, likely related to the inversion of the circadian melatonin secretion profile [10,13]. Behavioral manifestations in SMS may therefore be more closely associated with circadian organization and nocturnal awakenings than to sleep consolidation, as reflected by LSE alone. Another explanation may relate to the high prevalence of pharmacological treatments targeting sleep and behavioral symptoms in the present cohort. Indeed, almost all participants were receiving prolonged-release melatonin, often at relatively high doses, and more than half were also treated with psychostimulants or other psychotropic medications. Prolonged-release melatonin has been shown to improve sleep continuity and reduce nocturnal awakenings in children with SMS [13,30]. It is therefore plausible that treatment partially normalized sleep consolidation across the cohort, thereby reducing interindividual variability in LSE and masking potential associations with behavioral outcomes. In contrast, participants in the study by Yavuz-Kodat et al. were not receiving sleep-targeted pharmacological treatments, which may explain why the authors were able to detect an association between LSE and behavioral difficulties [18].

## 5. Conclusions

Overall, this exploratory study suggests that circadian rhythm disturbances, particularly markers of advanced circadian phase, are associated with specific behavioral difficulties in children with SMS. Given the observational design and limited sample size, these findings should be interpreted as hypothesis-generating rather than evidence of a causal relationship. If confirmed in larger prospective and interventional studies, these findings may help identify circadian rhythm characteristics as potential targets for future therapeutic interventions. Among chronotherapeutic approaches, light therapy could be investigated as a potential intervention for future studies aimed at delaying circadian phase in children with SMS. However, prospective controlled interventional studies are needed to determine whether such approaches improve both circadian organization and behavioral outcomes.

In this population characterized by frequent and prolonged nocturnal awakenings, L5 onset may represent a more reliable marker of circadian phase advance than sleep midpoint, as nocturnal awakenings occurring in the middle or latter part of the night are not taken into account when calculating sleep midpoint. These findings highlight the importance of distinguishing between sleep continuity and circadian rhythm organization when addressing behavioral difficulties in SMS.

Behavioral and environmental interventions targeting circadian phase represent promising avenues for future research. However, their potential to improve behavioral outcomes in children with SMS should be evaluated in prospective interventional studies.

## Figures and Tables

**Table 2:** Sleep and circadian rhythms measured according to the five behavioral domains evaluated with the Aberrant Behavior Checklist-Community (ABC-C) scale. Values are Spearman’s rank correlation coefficients.

	I-Irritability	II-Social Withdrawal	III-Stereotypic Behavior	IV-Hyperactivity	V-Inappropriate Speech
**Subjective sleep SDSC (n = 18)**					
Sleep disorders	0.181	0.453	0.472 *	0.438	0.373
Insomnia	0.472 *	0.701 **	0.648 *	0.425	0.381
Parasomnia	−0.322	−0.255	−0.289	0.232	0.109
Sleep breathing disorders	0.185	0.023	0.268	0.130	0.317
Non-restorative sleep	0.199	0.177	−0.081	0.248	0.476 *
Excessive daytime sleepiness	0.066	0.161	0.081	−0.037	−0.021
**H&O (n = 20)**	−0.565 *	−0.381	−0.209	0.009	0.052
**Objective sleep (ACT, n = 15)**					
Sleep start time (hh:mm)	−0.275	−0.267	−0.158	−0.290	−0.155
Sleep end time (hh:mm)	0.439	0.316	0.314	−0.013	0.141
TST (min)	0.357	0.210	0.147	0.018	0.301
SE (%)	−0.057	0.047	0.088	−0.175	0.341
SL (min)	0.132	0.099	−0.099	−0.046	0.049
WASO (min)	0.243	0.149	0.177	0.293	−0.173
Fragmentation (%)	0.011	0.305	0.430	0.068	−0.056
Number of wake episode	−0.139	−0.043	0.136	−0.220	−0.032
Average of wake episode	0.336	0.135	−0.077	0.329	−0.092
LSE (min)	0.368	0.362	0.219	−0.266	0.294
**Circadian rhythm (ACT, n = 13)**					
IS	−0.319	0.155	0.188	−0.181	0.331
IV	−0.187	−0.393	−0.287	−0.385	−0.519
RA	0.170	0.282	0.372	−0.027	0.144
L5-onset (hh:mm)	−0.396	−0.556 *	−0.543	−0.560 *	−0.517
M10-onset (hh:mm)	0.126	0.169	0.199	−0.066	−0.017
MSF (hh:mn)	0.007	0.061	0.036	−0.368	0.197
MSW (hh:mn)	0.182	0.046	0.002	−0.106	−0.076

ABC-C, Aberrant Behavior Checklist-Community; SDSC Sleep Disturbance Scale for Children; ACT, actigraphy; TST, Total Sleep Time; SE, Sleep Efficiency; SL, Sleep Latency; WASO, Wake After Sleep Onset; LSE, Longest Sleep Episode; IS, Interdaily Stability; IV, Intradaily Variability; RA, Relative Amplitude; L5-onset, start time of the 5 h period with the least movements; M10-onset, start time of the 10 h period with the highest amount of movement; MSF, Midpoint of Sleep on weekdays; MSW Midpoint of Sleep on weekend days. * *p* < 0.05; ** *p* < 0.01 using Spearman’s correlations.

## Data Availability

The original contributions presented in this study are included in the article. Further inquiries can be directed to the corresponding author.

## Abbreviations

The following abbreviations are used in this manuscript:

ABC-C	Aberrant Behavior Checklist-Community
ADHD	Attention-deficit/hyperactivity disorder
ASD	Autism Spectrum Disorder
CPRS-48	Conners’ Parent Rating Scale
DSM-5	Diagnostic and statistical manual of mental disorders—version 5
FRI	Fluid Reasoning Index
FSSA	Epworth Sleepiness Scale for Children and Adolescents Validated in French
H&O	Horne and Östberg Morningness–Eveningness Questionnaire
IDD	Intellectual Developmental Disorder
IQ	Intelligence Quotient
IS	Interdaily Stability
ISI	Insomnia Severity Index
IV	Intradaily Variability
MSF	Midpont of Sleep on Week Days
LSE	Longest Continuous Sleep Episode
MSW	Midpoint of Sleep on Weekend Days
N1	Stage 1
N2	Stage 2
N3	Stage 3
NPCRA	Non-Parametric Circadian Rhythm Analyses
PSG	Polysomnography
PSI	Processing Speed Index
RA	Relative Amplitude
RAI1	Retinoic Acid Induced 1
REM	Rapid Eye Movement
SDSC	Sleep Disturbance Scale for Children
SL	Sleep Latency
SMS	Smith–Magenis Syndrome
SP	Sleep Period
TSB	Time Spent in Bed
TST	Total Sleep Time
VABS	Vineland Adaptive Behavior Scales
VCI	Verbal Comprehension Index
VSI	Visuo-Spatial Index
WASO	Wakefulness After Sleep Onset
WISC	Wechsler Intelligence Scale for Children
WMI	Working Memory Index
WPPSI	Wechsler Preschool and Primary Scale of Intelligence
